# Critical Role of Heat Shock Protein 27 in Bufalin-Induced Apoptosis in Human Osteosarcomas: A Proteomic-Based Research

**DOI:** 10.1371/journal.pone.0047375

**Published:** 2012-10-16

**Authors:** Xian-biao Xie, Jun-qiang Yin, Li-li Wen, Zhen-hua Gao, Chang-ye Zou, Jin Wang, Gang Huang, Qing-lian Tang, Chiara Colombo, Wei-ling He, Qiang Jia, Jing-nan Shen

**Affiliations:** 1 Bone and Soft Tissue Tumor Center, The First Affiliated Hospital of Sun Yat-Sen University, Guangzhou, China; 2 The Institute of Biology, Guizhou Academy of Sciences, Guiyang, China; 3 Department of Anesthesiology, Sun Yat-Sen University Cancer Center, Guangzhou, China; 4 Department of Surgery, Istituto Nazionale Tumori, Milan, Italy; Boston University Medical School, United States of America

## Abstract

Bufalin is the primary component of the traditional Chinese herb “Chan Su”. Evidence suggests that this compound possesses potent anti-tumor activities, although the exact molecular mechanism(s) is unknown. Our previous study showed that bufalin inhibited growth of human osteosarcoma cell lines U2OS and U2OS/MTX300 in culture. Therefore, this study aims to further clarify the *in vitro* and *in vivo* anti-osteosarcoma effects of bufalin and its molecular mechanism of action. We found bufalin inhibited both methotrexate (MTX) sensitive and resistant human osteosarcoma cell growth and induced G2/M arrest and apoptosis. Using a comparative proteomics approach, 24 differentially expressed proteins following bufalin treatment were identified. In particular, the level of an anti-apoptotic protein, heat shock protein 27 (Hsp27), decreased remarkably. The down-regulation of Hsp27 and alterations of its partner signaling molecules (the decrease in p-Akt, nuclear NF-κB p65, and co-immunoprecipitated cytochrome c/Hsp27) were validated. Hsp27 over-expression protected against bufalin-induced apoptosis, reversed the dephosphorylation of Akt and preserved the level of nuclear NF-κB p65 and co-immunoprecipitated Hsp27/cytochrome c. Moreover, bufalin inhibited MTX-resistant osteosarcoma xenograft growth, and a down-regulation of Hsp27 *in vivo* was observed. Taken together, bufalin exerted potent anti-osteosarcoma effects *in vitro* and *in vivo,* even in MTX resistant osteosarcoma cells. The down-regulation of Hsp27 played a critical role in bufalin-induced apoptosis in osteosarcoma cells. Bufalin may have merit to be a potential chemotherapeutic agent for osteosarcoma, particularly in MTX-resistant groups.

## Introduction

Osteosarcoma is the most common primary malignant bone tumor in young people, with an annual incidence of 5.6 per million [1]. The survival rate of osteosarcoma patients has improved with advances in chemotherapeutic agents, and the five-year survival rate has reached 60–70% [2], [3]. Currently used chemotherapy regimens are based on a combination of MTX, doxorubicin, cisplatin, and ifosfamide, of which MTX is the most active [4]. Nevertheless, a considerable number of patients are either not sensitive to chemotherapy or develop drug resistance [3]. One strategy to overcome this problem is to find alternative anti-cancer agents that will increase drug-response rates, avoid chemo-resistance, and improve clinical outcomes [5], [6], [7].

Bufalin, a bufadienolide, is the primary component extracted from the traditional Chinese herb “Chan Su” [8] and has been used as an effective cardiotonic drug. Recently, significant anti-tumor activity of bufalin in several cancer cells has been reported [9]–[15]; however, very little is known about its effects on osteosarcoma cells. Previously, we demonstrated that bufalin had significant *in vitro* anti-tumor activity on the U2OS and U2OS MTX300 cell lines [16]. However, the molecular basis of the anti-osteosarcoma activity and the *in vivo* effects of bufalin in osteosarcoma remain unclear. As such, the aim of the current study was to confirm the *in vitro* and *in vivo* anti-osteosarcoma effects of bufalin and to determine its mechanism of action to evaluate its potential as an alternative drug in the treatment of osteosarcoma patients.

## Materials and Methods

### Drugs and Reagents

Bufalin, MTT (methyl thiazolyl tetrazolium), and hoechst 33258 were purchased from Sigma Chemical Co. (St Louis, MO, USA), while MTX was obtained from the National Institute for the Control of Pharmaceutical and Biological Products (Beijing, China). Additionally, Hsp27, phospho-Akt, Akt, NF-κB p65, cytochrome c, and GAPDH antibodies were purchased from Cell Signal Technologies (Danvers, MA, USA), and the antibodies for α-tubulin, lamin A/C, PARP and cleaved-PARP were obtained from Santa Cruz Biotechnology (Santa Cruz, CA, USA).

### Cell Lines

The human osteosarcoma cell lines U-2OS, U-2OS/MTX300, SaOS-2, and IOR/OS9 were gifts from Dr. M. Serra (Istituti Ortopedici Rizzoli, Bologna, Italy). U-2OS and SaOS-2 were all obtained from the American Type Culture Collection (Rockville,MD). U-2OS/MTX300 and IOR/OS9 were established at the Laboratorio di Ricerca Oncologica, Istituti Ortopedici Rizzoli, (Bologna, Italy) and previously characterized [17], [18]. The U-2OS MTX300-resistant variant was continuously cultured in the presence of 300 µg/L MTX [18]. All the other cells were cultured in Dulbecco’s modified Eagle’s medium (DMEM) (Gibco, Grand Island, NY, USA) and supplemented with 10% fetal calf serum (Hyclone, Logan, UT, USA), penicillin (10,000 U/L), and streptomycin (100 mg/L) at 37°C in a 5% CO2 humidified incubator.

### MTT Assay

A total of 2,000 cells were plated in 96-well flat-bottom plates and exposed to bufalin at different concentrations in a final volume of 180 uL. At the indicated times, 20 µL of 5 mg MTT/ml in PBS were added to each well, and the plates were incubated for 4 h. After removal of the medium, 150 µL DMSO was added to each well to dissolve the formazan crystals. Absorbance at 490 nm was determined using a microplate reader.

### Cell Cycle Analysis

Approximately 2×10^6^ cells were treated with bufalin at 37°C in a 5% CO2 incubator for 24 h. The cells were collected and analyzed with a flow cytometer (Becton Dickinson) after propidium iodide staining.

### Hoechst 33258 Staining

After 48 h in the indicated concentrations, the bufalin-treated cells were washed twice with PBS and stained with the DNA-specific dye hoechst 33258 (10 mL, 10 mg/L). The cells were then incubated at 37°C for 10 min and washed with PBS for morphological observation using an Olympus photomicroscope with an epifluorescence attachment (Tokyo, Japan).

### Annexin V/PI Staining Assay for Apoptosis

The cells were treated with 50 nM bufalin for 48 h. The cells were then collected, washed twice with PBS, resuspended at a density of 1×10^6^ cells/ml, and incubated with Annexin V-FITC and propidium iodide for 30 min in the dark. Cell apoptosis was analyzed using FACScan (Becton Dickinson, Franklin Lakes, NJ, USA), and the data were analyzed via CellQuest software (Becton Dickson, Franklinn Lakes, NJ, USA).

### Two-dimensional Gel Electrophoresis

Two-dimensional gel electrophoresis (2DE) was conducted with Amersham Biosystems IPGphor IEF and Hoefer Tank (13 cm) units in accordance with a previously described protocol [19], [20]. Protein samples (250 µg), extracted from untreated controls and bufalin-treated cells, were used for 2DE analysis. All gels were visualized by silver staining.

### Image Analysis and Mass Spectrometry (MS) Peptide Sequencing

Image acquisition and analysis were performed with Image Scanner (Amersham Biosciences) and Image Master 2D Platinum software (Amersham Biosciences). The gel images of bufalin-treated cells were compared with untreated controls. Altered protein spots with consistent and significant volume changes (>1.5-fold difference) were selected for MALDI-TOF MS and tandem mass spectrometric analysis with a 4700 proteomics analyzer (“TOF/TOF” Applied Biosystemes Inc., Foster City, CA). The resulting data were processed using the 4700 Explorer software.

### Preparation of Protein Nuclear Extracts

Nuclear extracts were prepared using the Nuclear Extract Kit (Active Motif, Carlsbad, CA), according to the manufacturer’s instructions. The protein levels of NF-κB p65 in the nucleus were determined by western blot analysis.

### Immunoprecipitation

The cells were lysed with an immunoprecipitation buffer (50 mM HEPES [pH 7.6], 150 mM NaCl, 5 mM EDTA, and 0.1% NP-40). Each supernatant was centrifuged for 15 min at 15,000×g and incubated with a cytochrome c antibody (1∶100) on a rotator at 4°C overnight. Then, protein A-sepharose was used to precipitate the immunocomplexes, and the pellet was analyzed by western blotting.

### Western Blot Analyses

The cells were treated with bufalin at the indicated doses. The western blots were performed as previously described [21] to detect the expression levels of target proteins.

### RT-PCR Analysis

Total RNA was extracted by using the TRIzol reagent, according to the manufacturer’s protocol. The RT-PCR analysis were performed as previously described [16] to detect the expression mRNA level of Hsp27. For HSP27, the forward primer was 5′-TGGACCCCACCCAAGTTTC-3′ and reverse primer was 5′-CGGCAGTCTCATCGGATTTT-3′, and for beta actin, the forward primer was 5′-AGCGAGCATCCCCCAAAGTT-3′ and reverse primer was 5′-GGGCACGAAGGCTCATCATT -3′.

### Transient Transfection

pCMV6-AC-Hsp27 and pCMV6-AC plasmids were purchased from OriGene Technologies (Rockville, MD, USA). The cells were plated into 6-well plates (2×10^5^ cells/well) before transfection with Lipofectamine 2000 (Invitrogen, Carlsbad, CA, USA), according to the manufacturer’s instructions. The plasmid DNA and transfection reagent were prepared at a ratio of 1∶2 and incubated with the cells for 24 h prior to incubation with bufalin.

### Animal Studies

Female nude BALB/c mice, 6 weeks old, were purchased from the Experimental Animal Center of Guangdong Province, China. All studies were conducted in accordance with Center of Experiment Animal of Sun Yat-sen University guidelines and were specifically approved by ethics committee of Center of Experiment Animal of Sun Yat-sen University. The tumors were established via subcutaneous injection of 5×10^6^ U2OS/MTX300 cells into the axilla of the mice. The tumor volumes were estimated according to the formula π/6×a^2^×b, where *a* is the short axis, and *b* the long axis. Ten days after injection, the mice were randomly divided into 4 groups (6 mice per group). The control group received daily intraperitoneal (ip) injections of 100 µl of vehicle, while the MTX group received ip injections of MTX (250 mg/kg) with calcium leucovorin rescue (24 mg/kg at 16, 20, or 24 hours after MTX) per week [22]. The bufalin treatment groups received ip injections of either 0.75 or 1.5 mg/kg per day [23]. When the tumor size in the control group reached 1.5 cm, all mice were sacrificed, and the tumors were removed.

### Statistical Analyses

All data were derived from at least three independent experiments, and the results were expressed as the mean ± standard error. The differences were assessed using Student’s *t*-test or one-way ANOVA, and a *p-*value of <0.05 was considered significant. All analysis was performed by SPSS 13.0 (SPSS Inc, Chicago, IL, USA).

## Results

### Bufalin Exerts Potent Anti-osteosarcoma Effects *in vitro*

**Figure 1:**
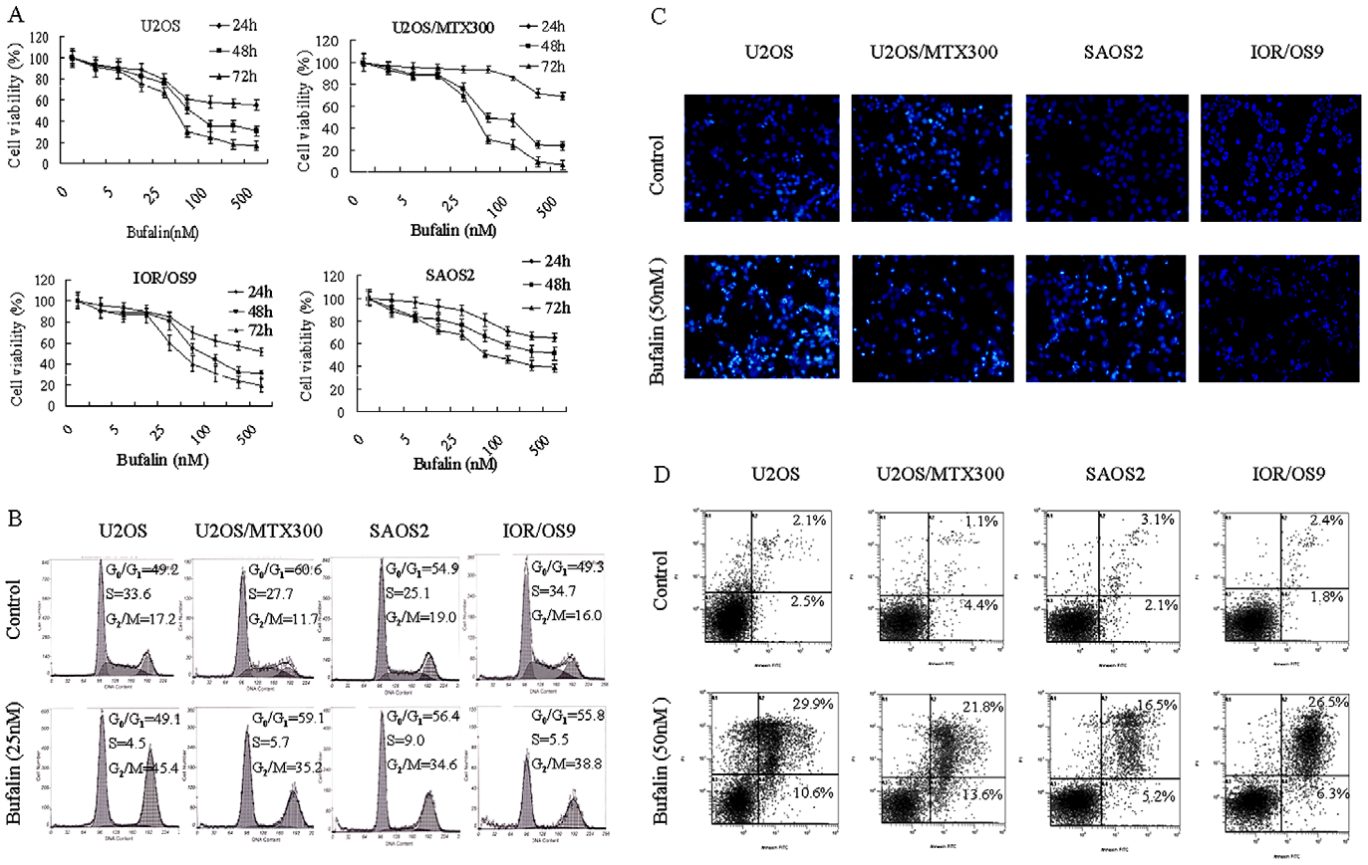
Bufalin exerted potent anti-osteosarcoma activity *in vitro*. (A) Bufalin induced a dose- and time-dependent decrease in osteosarcoma cell growth. (B) Bufalin treatment resulted in G_2_/M cell cycle arrest in osteosarcoma cell lines (PI staining and FACS analysis). (C) Hoechst staining showed typical apoptotic morphology changes after bufalin treatment. Each image was taken at a magnification of 100×. (D) Annexin-V/PI staining and FACS analysis demonstrated a significant increase in apoptosis induced by bufalin in osteosarcoma cells.

To identify the effects of bufalin on human osteosarcoma cell lines, we firstly tested various doses of bufalin on the viability of the human osteosarcoma cell lines U-2OS, U-2OS/MTX300, SaOS-2, and IOR/OS9 in different time points. Bufalin exerted a time- and dose-dependent inhibition to all osteosarcoma cell lines (Figure 1A). These initial results suggested that bufalin inhibited the viability of various human osteosarcoma cells *in vitro*, which includes the MTX resistant cell line U2-OS/MTX300. To detect the effects of bufalin on cell cycle distribution, flow cytometry was performed after treating the cells with bufalin (25 nM for 24 h). An evident G_2_/M phase arrest was observed in all cell lines (Figure 1B). Hoechst 33258 staining was used to detect morphological changes after bufalin treatment. All cells were treated with bufalin (50 nM for 48 h). Fluorescent staining showed classical apoptosis in all cell lines, including volume reduction, chromatin condensation, nuclear fragmentation, and the appearance of apoptotic bodies (Figure 1C). Annexin V/PI staining was then used to assess the apoptotic rate. The percentage of apoptotic cells increased noticeably after bufalin treatment in U-2OS, U-2OS/MTX300, SaOS-2, and IOR/OS9 cells (Figure 1D).

### Bufalin Modifies the Expression of Proteins Involved in Cellular Metabolism, Apoptotic Processes and Cytoskeleton Structure

**Figure 2:**
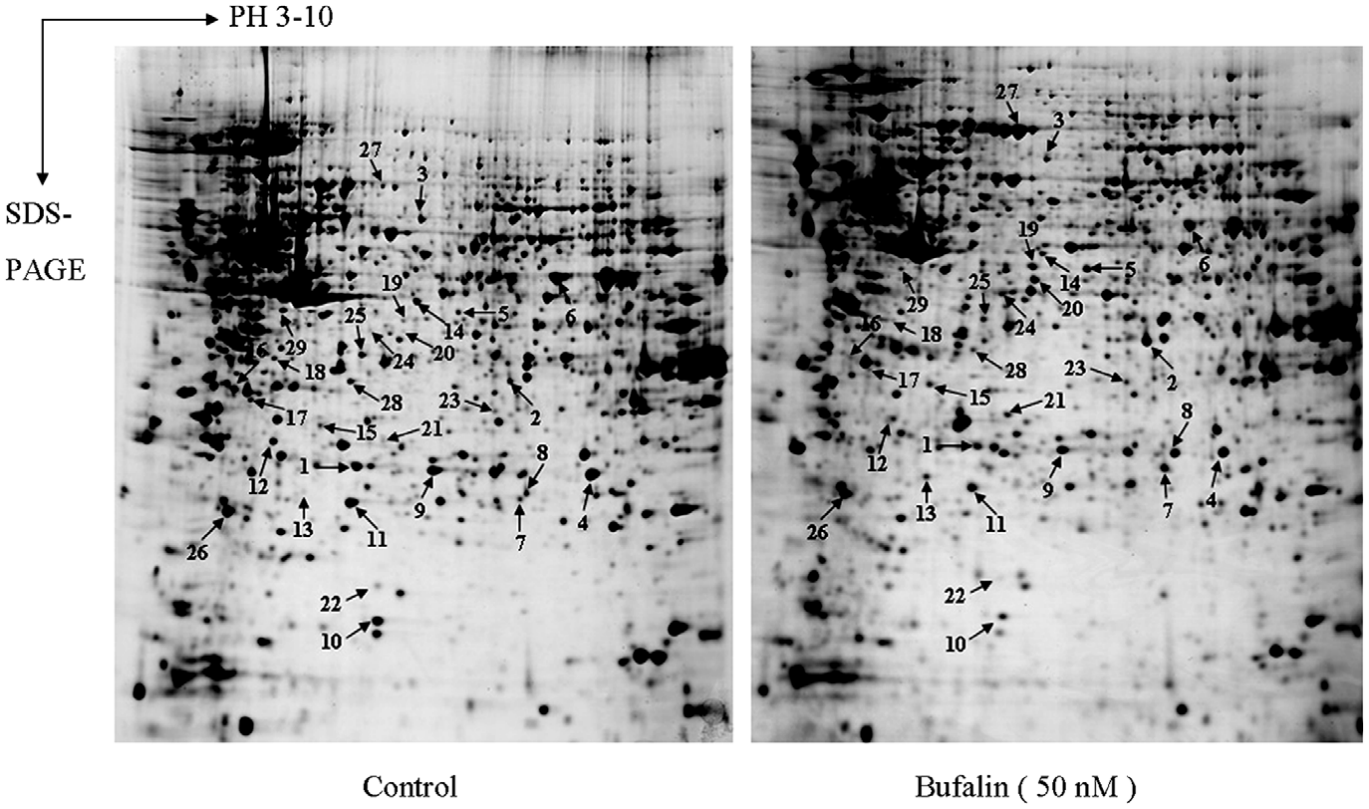
Representative images of 2DE gels showing differentially expressed proteins following bufalin treatment.

**Figure 3:**
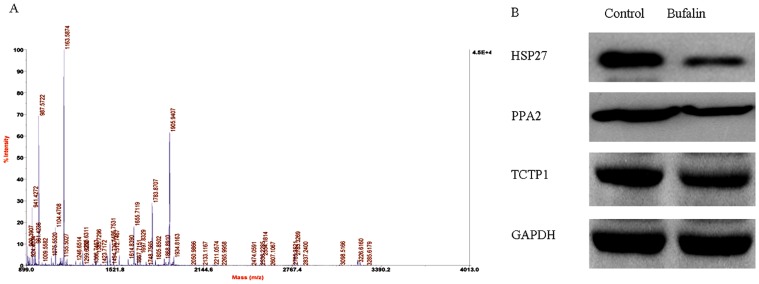
Identification and verification of candidate proteins by MALDI-MS and western blot analyses. (A) A typical MALDI-MS spectrum of spot #10 from the 2-DE map; this candidate protein was identified as Hsp27. (B) Western blot analyses were used to validate the changes in apoptosis-related proteins following bufalin treatment.

Proteomic technology was applied to study the regulation of proteins that might play important roles in multiple signaling pathways following bufalin treatment. The osteosarcoma cell line U2OS was treated with bufalin (50 nM for 48 h). 2DE and MALDI-TOF/TOF MS mediated protein identification were performed. Through image analysis, an average of 1,053 protein spots were found on each gel. For 29 protein spots, the difference between the two gels was more than 1.5-fold (*p*<0.05 for all spots; see Figure 2). With MALDI-TOF/TOF MS, 26 of the 29 protein spots were successfully identified. A representative MS map for the protein identification is shown in Figure 3A. Some spots corresponded to the same protein (i.e., spots #1 and #10 corresponded to Hsp27, and spots #19 and #20 corresponded to the ATCB protein; Table 1). Therefore, for 24 spots, a unique protein was identified (Table 1). Among these, 8 proteins were up-regulated and 16 were down-regulated. According to their function, 12 proteins were related mainly to cellular metabolism (e.g., energy production, RNA processing, and protein synthesis). Interestingly, 11 of the 12 metabolic proteins were down-regulated, while only one was up-regulated after bufalin treatment; the up-regulated protein was identified as DnaJ homolog, subfamily B, member 11, a molecular chaperone that has anti-oxidative activity. Furthermore, the expression of three proteins involved in apoptotic processes, changed; in particular, Hsp27, protein phosphatase 2 (PPA2), and tumor protein translationally-controlled 1 (TPTC1) were all down-regulated and validated by western blot analysis (Figure 3B). Among the apoptosis-related proteins, Hsp27 showed the greatest change (3.65 fold). Beta actin, gamma 1 actin, cytokeratin 18 and ACTB proteins, which are involved in cytoskeleton structure, were up-regulated. A decrease in the expression of the epsilon subunit of coatomer protein complex isoform (COPE), a carrier protein, was detected. Finally, we observed a modification in the expression of PRO2619 and tropomodulin 3, for which a clear function has not yet been defined.

**Table 1:** Identification and classification of differential protein levels in bufalin-treatment and control cells using MALDI-TOF/TOF MS.

Spot No.	Protein ID	NCBI accession No.	Protein MW (Da)	Protein PI	Protein score/CI (%)	Fold changes(bufalin: ctrl)	Regulation
	**A)Energy metabolism**						
4	triosephosphate isomerase 1 isoform 1	gi|4507645	26652.7	6.45	900/100	2.02385925	down
6	enolase 1	gi|4503571	47139.3	7.01	551/100	2.348275148	down
	**B)RNA processing**						
7	heterogeneous nuclearribonucleoprotein H1	gi|5031753	49198.4	5.89	427/100	2.149929929	down
14	TAR DNA binding protein	gi|6678271	44711.3	5.85	309/100	1.670435594	down
16	heterogeneous nuclearribonucleoprotein K	gi|55958547	41780.7	5.43	607/100	1.835848704	down
22	deoxyuridine triphosphataseisoform 2	gi|4503423	17736.9	6.15	518/100	3.783149115	down
	**C)Protein metabolic process**						
3	T-complex protein 1 isoform a	gi|57863257	60305.6	5.8	659/100	1.631723574	down
5	DnaJ (Hsp40) homolog, subfamily B,member 11	gi|7706495	40488.6	5.81	538/100	3.029160132	up
11	Chain A, Crystal Structure Of HumanGlutathione	gi|2554831	23327	5.43	717/100	1.929768696	down
12	PSMA3	gi|48145983	28397.1	5.19	542/100	1.71329061	down
21	laminin-binding protein	gi|34234	31773.9	4.84	336/100	2.300769582	up
23	cytokine induced protein 29 kDa	gi|32129199	23656.4	6.1	324/100	2.563022416	up
25	Ribosomal protein, large, P0	gi|12654583	34252.8	5.42	626/100	1.982930034	down
	**D)Phosphate metabolism**						
28	pyrophosphatase 1	gi|11056044	32639.2	5.54	585/100	1.877354871	down
	**E)Apoptosis related protein**						
1,10	heat shock protein 27	gi|662841	22313.3	7.83	589/100	3.651903817	down
18	protein phosphatase 2 (formerly 2A),catalytic subunit	gi|54695922	35582.3	5.21	117/100	2.106859386	down
26	Tumor protein, translationally-controlled 1	gi|15214610	19644.6	4.84	526/100	1.635466251	down
	**F)Cytoskeleton organization**						
8	cytokeratin 18	gi|30311	47305.2	5.27	587/100	5.813800354	up
15	actin, gamma 1	gi|16924319	40477.2	5.78	576/100	9.184324537	up
19,20	ACTB protein	gi|15277503	40194.1	5.55	604/100	5.564858518	up
24	actin, beta	gi|14250401	40978.4	5.56	549/100	5.564156789	up
	**G)Transport**						
17	epsilon subunit of coatomer protein complexisoform a	gi|31542319	34460.3	4.97	434/100	3.285326325	down
	**H)Unknown**						
27	PRO2619	gi|11493459	56745.2	5.96	181/100	9.667976149	up
29	tropomodulin 3	gi|57997483	39530.2	5.08	554/100	2.478153496	down

### Bufalin Modifies the Endogenous Level of Hsp27 and its Relevant Molecules, p-AKT, Nuclear NF-κB p65 and Cytochrome c, in Osteosarcoma Cells

**Figure 4:**
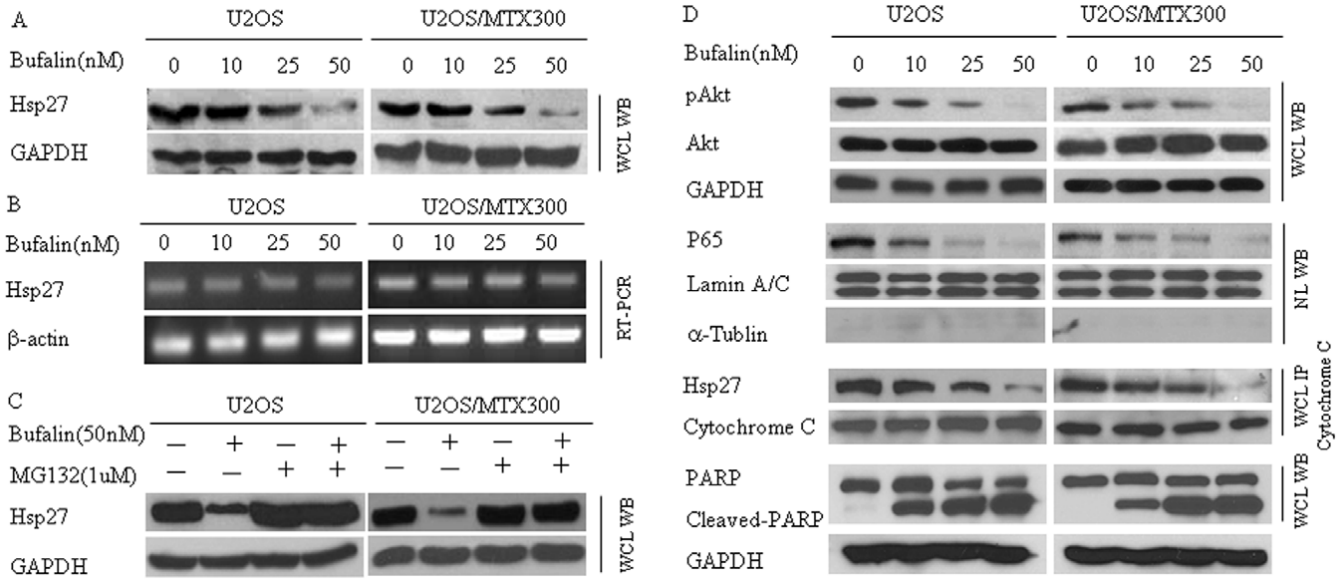
Bufalin modifies the endogenous level of Hsp27 and its relevant molecules. (A) The level of Hsp27 in U-2OS and U-2OS/MTX300 cells were detected by Western blot after bufalin treatment. (B) Following incubation with bufalin for 48 h, the level of Hsp27 mRNA was examined by RT-PCR analysis. (C) Cells were submitted to bufalin treatment with or without MG132 incubation. Levels of Hsp27 was revealed by immunobloting. (D) Bufalin treatment decreased the expression levels of p-Akt, nuclear NF-κB P65, and the levels of co-immunoprecipitated cytochrome c/Hsp27. In addition, the levels of cleaved-PARP were significantly increased. WCL: whole cell lysate, NL: nuclear lysate, IP: immunoprecipitation.

Previous studies have shown that Hsp27 is an important anti-apoptotic protein. In the present research, it was found to yield the most evident modification following bufalin treatment. Therefore, we paid particular attention to this protein. Dose-dependent down-regulation of Hsp27 after bufalin treatment was confirmed by western blot analysis in U2OS and U2OS/MTX300 cells (Figure 4A). To get a hint how bufalin affects Hsp27, we used RT-PCR to detect the Hsp27 mRNA level. As shown in Figure 4B, Hsp27 mRNA level was not significantly altered after treated with bufalin, while the level of Hsp27 protein was reduced (Figure 4A). These results showed that bufalin could decrease the Hsp27 protein level without modulating the expression of its mRNA transcripts levels. In order to measure effects of bufalin on degradation of Hsp27 protein, cells where treated with the proteasome inhibitor MG132. And we found that MG132 up-regulated the level of Hsp27 when treated with bufalin (Figure 4C). This suggests that an increased degradation of Hsp27 occurs in osteosarcoma cells treated with bufalin.

Recently, Hsp27 has been demonstrated to regulate apoptosis via interactions with key molecules of the cell-death signaling pathway [24]–[30]. As shown in Figure 4D, bufalin treatment induces a decrease in p-Akt, nuclear NF-κB p65, and co-immunoprecipitated Hsp27/cytochrome c. Furthermore, total PARP (the substrate of caspase-3) decreased, while cleaved-PARP remarkably increased. These data indicate that Hsp27 and its partner molecules may play an important role in bufalin-induced apoptosis in osteosarcoma cells.

### Ectopic Expression of Hsp27 Partially Protects Cells against Bufalin-induced Apoptosis

**Figure 5:**
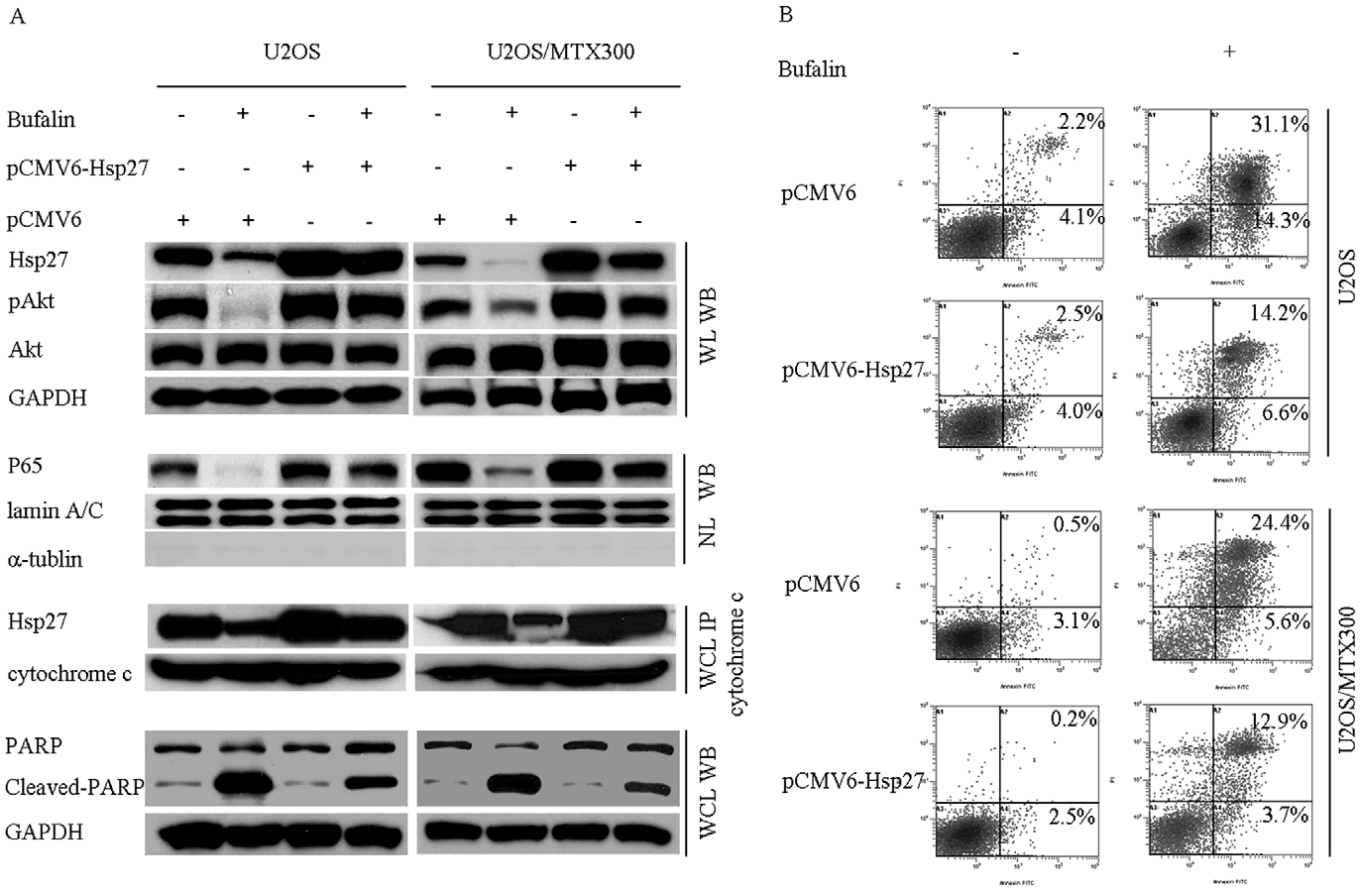
Overexpression of Hsp27 partially protected the cells against bufalin-induced apoptosis. (A) pCMV6-AC-Hsp27 and control vector-transduced U-2OS cells were treated with or without bufalin, and Hsp27, p-Akt, Akt, nuclear NF-κB P65, PARP, cleaved-PARP and co-immunoprecipitated cytochrome c/Hsp27 were detected by western blot analyses. (B) Annexin-V/PI staining FACS analysis demonstrated a significant decrease in the apoptotic rate in cells that over-expressed Hsp27.

To confirm the role of Hsp27 in bufalin-induced apoptosis, U2OS cells were transfected with pCMV6-AC-Hsp27 or pCMV6-AC and incubated with or without bufalin (50 nM) for 48 h. As shown in Figure 5A, over-expression of Hsp27 increased the levels of p-Akt and abolished the bufalin-induced dephosphorylation of Akt. Although nuclear NF-κB p65 levels did not notably increase with overexpression of Hsp27, there may be a possible protection against the down-regulation of nuclear NF-κB p65 following bufalin treatment. Moreover, levels of co-immunoprecipitated Hsp27/cytochrome c preserved, and a decrease in cleaved-PARP was observed (Figure 5A). Finally, a significant decrease in the apoptotic rate was found in cells that over-expressed Hsp27, as compared to the controls (Figure 5B). These data indicate that Hsp27 may play a critical role in bufalin-induced apoptosis.

### *In vivo* Anti-osteosarcoma Activity of Bufalin

**Figure 6:**
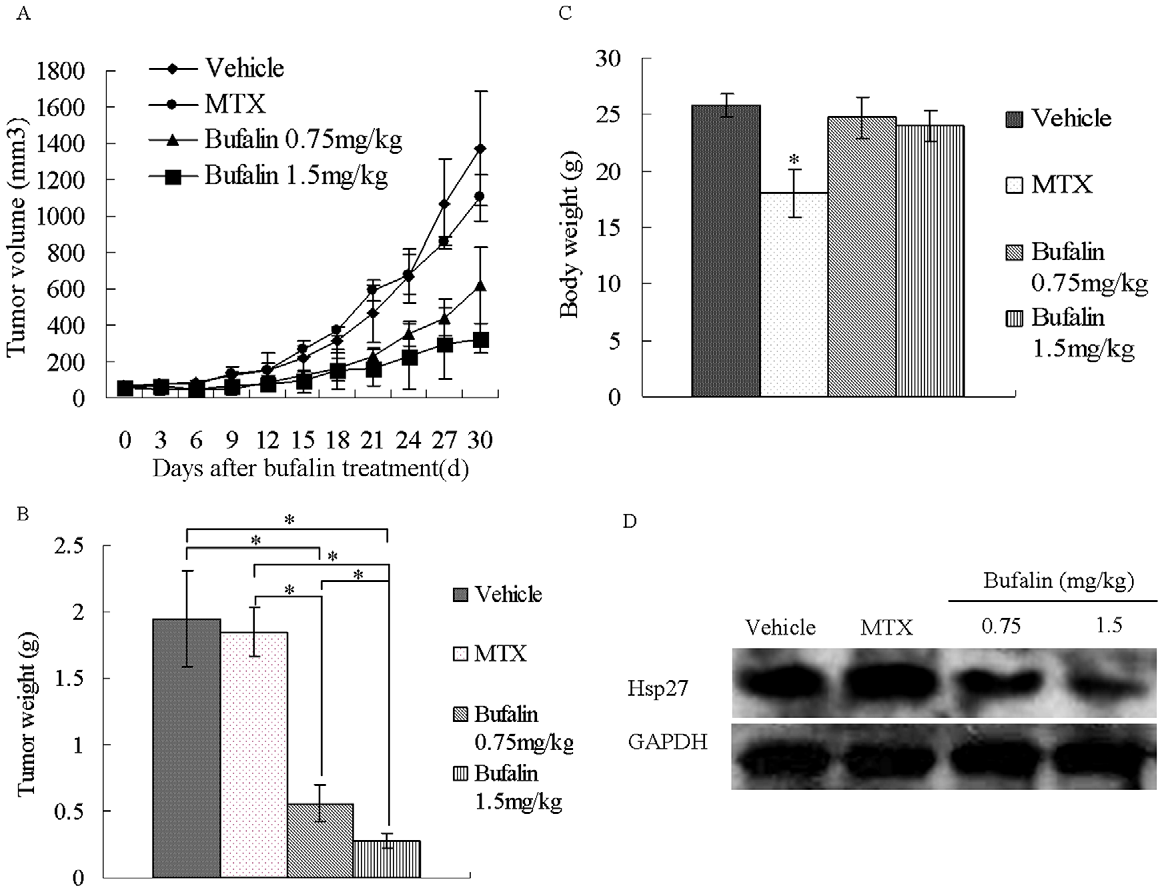
Bufalin exhibited strong anti-osteosarcoma activity *in vivo*. Mice with U-2OS/MTX300 xenografts were treated with bufalin, MTX or vehicle control (n = 6). (A) Bufalin-treated mice exhibited slower tumor growth. (B) A significantly decreased tumor weight at study termination, as compared to control or MTX-treated mice, was observed. (C) The average body weight of the mice in the bufalin groups did not decrease significantly. (D) Western blot analyses confirmed decreased Hsp27 expression in bufalin-treated tumors. All values are presented as the mean ± SD, ^*^p<0.05.

The *in vitro* data prompted us to test the anti-osteosarcoma activity of bufalin in mice harboring a subcutaneous xenograft of U2OS/MTX300 cells. Treatment with 0.75 and 1.5 mg/kg bufalin induced significant tumor growth inhibition (see Figure 6A), as compared to the control and MTX treatment groups (*p*<0.05). The average tumor weights at the termination of the study were as follows: control group, 1.95±0.36 g; MTX group, 1.85±0.19 g; 0.75 mg/kg bufalin group, 0.56±0.14 g; and 1.5 mg/kg bufalin group, 0.28±0.06 g (Figure 6B). At the conclusion of the *in vivo* study, the average body weight of the mice in the two bufalin and control groups was not significantly different, whereas the MTX group showed a remarkable decrease in body weight. These data indicate that there was little to no toxicity of bufalin at doses of 0.75 mg/kg and 1.5 mg/kg under the same treatment conditions (Figure 6C). Western blot analyses were performed to detect the expression levels of Hsp27 in the xenograft tumors. The results revealed a decrease in the expression of Hsp27 in the bufalin treatment groups, while an increase was noted in the MTX group, as compared to the vehicle (Figure 6D).

## Discussion

Various chemotherapeutic agents, such as MTX, doxorubicin, cisplatin, etoposide, and isophosphamide, have been used either alone or in combination for the treatment of osteosarcoma [1], [3]. However, the efficacy of chemotherapy is limited, and drug resistance is a common clinical problem that can significantly diminish clinical outcomes [1], [5]. Therefore, the development of novel anti-osteosarcoma agents remains a challenge in the treatment of this disease; one strategy for overcoming this challenge is to seek new pharmaceuticals of natural origins [6], [7].

Bufalin is the most active component of the traditional Chinese herb “Chan Su”, which is obtained from the skin and parotid venom glands of toads. Recently, bufalin has been shown to inhibit cell proliferation and induce apoptosis in different cancers including leukemia [9], [10], [11], prostate cancer [12], breast cancer [13], lung cancer [14] and hepatocellular carcinoma [15]. As such, several mechanisms of action have been proposed, including the suppression of Topo II and Na+, K+ -ATPase [9], activation of AP-1 [10], Rac1 [11], cdc2 kinase and casein kinase II [9], induction of Tiam1 [11], and increase of intracellular calcium concentrations [12]. Bufalin also down-regulates the apoptosis-related protein Bcl-2 and up-regulates Bax and p21 [16]. Moreover, bufalin inhibits the STAT3/Mcl-1 pathway [13] and the VEGFR1/VEGFR2/EGFR/cMet-Akt/Erk/p38MAPK-NF-κB signaling pathways [14] and activates Fas and mitochondria-mediated pathways to trigger apoptosis [15].

In the present research, we found that bufalin could strongly inhibit the viability of all four types of human osteosarcoma cell lines in a dose- and time-dependent manner, including in the MTX resistant cell line U2OS/MTX300. Furthermore, the induction of a G2/M cell cycle arrest and apoptosis were also observed. These results indicate that the anti-tumor activity of bufalin in osteosarcoma cells is due to a G2/M arrest and apoptosis, which is consistent with previous studies [10]–[16].

To determine the potential molecular mechanism of action of bufalin, we used a proteomic-based screening and found that the expression levels of 24 proteins changed following bufalin treatment; these proteins were primarily related to cellular metabolism, apoptosis, and cytoskeleton organization. Specifically, 12 proteins were involved in the metabolic process, including energy production, RNA processing, and protein synthesis and phosphate assembly and degradation. Furthermore, the current data showed that most of these proteins were down-regulated, which indicates that bufalin treatment could reduce cellular metabolism. Three apoptosis-related proteins were also identified. Of these proteins, PP2A has phosphatase activity and has important functions in cell proliferation and death, cell mobility, cell cycle, and the regulation of various signaling pathways. Additionally, PP2A might be a critical tumor suppressor [31] . However, in this study, PP2A was down-regulated following bufalin incubation, which could only be a compensatory change. TCTP1 plays a role in the regulation of cell growth and apoptosis, and its anti-apoptotic activity is related to its interaction with Mcl-1, Bcl-XL, and p53 [32] . Although TCTP1 was down-regulated following bufalin treatment, the most dramatically altered protein was Hsp27 (3.65-fold down-regulation). Therefore, our study focused on this molecule.

Hsp27 is a member of the heat shock protein family, and many studies have demonstrated that Hsp27 is able to block the apoptotic process that interacts with different molecules [24], [33]. Hsp27 effectively blocks caspase-dependent apoptotic pathways. The small heat shock protein binds to cytochrome c, thus inhibiting the formation of the apoptosome [25], [26]. However, Hsp27 also regulates some upstream signaling pathways. As such, the interaction between Hsp27 and Akt is necessary for the activation of Akt in stressed cells [27], [28]. HDAC6, STAT2 and procaspase-3 are also client proteins of Hsp27 [33]. Recent findings have indicated that Hsp27 might protect cells from etoposide or TNF-α-induced apoptosis by increasing the activity of NF-κB [29], [30]. Other mechanisms have been described for the anti-apoptotic activity of Hsp27, including the binding of F-actin to prevent disruption of the cytoskeleton [26] and interacting with and inhibiting the Daxx apoptotic pathway [34]. Hsp27 also plays an important role in tumorigenesis, and several studies have demonstrated that Hsp27 increases the metastatic potential of tumor cells and resistance to therapy [35]. Hsp27 was also found to be over-expressed in a variety of human tumors, including osteosarcoma [36], [37] and has been shown to correlate with tumor metastasis and poor prognosis [37], [38]. Because of its anti-apoptotic and tumorigenic properties, Hsp27 could be a potential therapeutic target for osteosarcoma.

In the present study, endogenous level of Hsp27 were found significantly reduced with bufalin concentration. While the expression levels of its mRNA was not changed remarkably. Of interest, we found that the proteasome inhibitor MG132 incubation could restore the Hsp27 level following bufalin treatment. Therefore, bufalin treatment could probably induce degradation of Hsp27 in osteosarcoma cells. We also observed significant alteration of its partner signaling molecules. Specifically, Akt was dephosphorylated, nuclear NF-κB p65 levels were decreased, the Hsp27/cytochrome c interaction was reduced, and PARP was cleaved. Moreover, the over-expression of Hsp27 could reverse the effects of bufalin-treatment on Akt, NF-κB, cytochrome c, and PARP and partially rescue cells from bufalin-induced apoptosis. Taken together, these observations suggest that Hsp27 may play an important role in bufalin-induced apoptosis.

The *in vivo* experiments were conducted via the xenograft model of nude mice with the MTX-resistant human osteosarcoma cell line U2OS/MTX300. The doses were chosen according to previous data that indicated low toxicity of these doses [23]. The results showed that bufalin was able to obviously reduce the volume and weight of tumors compared to the control and MTX groups. Although bufalin possessed potent *in vivo* anti-tumor activity, the toxicity of the drug was reduced compared to the classic chemotherapy drug MTX. Furthermore, western blot analysis confirmed the inhibition of Hsp27 expression after bufalin treatment, supporting the *in vitro* results.

In the present study, we demonstrated that bufalin has a satisfactory anti-tumor activity through the induction of apoptosis and G_2_/M phase arrest in different human osteosarcoma cell lines and a potent anti-tumor effect in the osteosarcoma xenograft model. These findings indicate that bufalin may be useful as an alternative chemotherapeutic agent in osteosarcoma patients, particularly in MTX-resistant groups. Finally, while the mechanism by which bufalin-induced apoptosis was possibly mediated, at least in part due to down-regulation of Hsp27, further studies are still needed to clarify the exact role of Hsp27 in bufalin-induced apoptosis.
